# Association of low vitamin B_12_ levels with depressive and schizophrenia spectrum disorders in child and adolescent psychiatric inpatients

**DOI:** 10.1007/s00787-025-02662-4

**Published:** 2025-03-18

**Authors:** Gerard Anmella, Eva Varela, Nuria Prades, Anna Giménez-Palomo, Laura Espinosa, Clara de Castro, Ramon Deulofeu, Mireia Solerdelcoll, Ástrid Morer, Inmaculada Baeza

**Affiliations:** 1 Hospital Clínic de Barcelona, Barcelona, Spain; 2 Centro Educativo Terapéutico Mentalia Área Norte, Madrid, Spain; 3 Hospital universitario La Plana, Villarreal, Spain; 4 Fundació Althaia, Manresa, Spain; 5 CSMIJ Granollers, Hospital Sant Joan de Déu, Granollers, Spain; 6 Centro de Investigación Biomédica en Red de Enfermedades Hepáticas y Digestivas, Madrid, Spain; 7 Neuroscience Institute, deoartment of Medicine, University of Barcelona, Barcelona, Spain; 8 Centro de Investigación Biomédica en Red de Salud Mental, Madrid, Spain

**Keywords:** Vitamin, Folate, Cobalamin, Depression, Child and adolescent, Psychiatry

## Abstract

Folate and vitamin B_12_ are associated with neurodevelopment and neurotransmitter synthesis and insufficiencies of these nutrients could be linked to psychiatric disorders in children and adolescents. To assess serum levels of folate and B_12_ in child and adolescent psychiatric inpatients and examine possible links between these levels and different psychiatric disorders. Child and adolescent psychiatric inpatients admitted in a general hospital during a 3-year period were included for analysis. Folate and B_12_ levels were measured when the subjects were admitted. Psychiatric diagnoses were made following DSM-5 criteria and grouped into categories. Logistic regression analysis was used to study the effects of socio-demographic variables as well as folate and B_12_ levels, insufficiencies and deficits as possible predictors of outcome (psychiatric diagnostic category). 729 inpatients (60.6% female, mean age: 15.1 ± 2 years) were included. A total of 42.9% presented insufficient folate levels and 19.4% insufficient B_12_ levels. Insufficient B_12_ levels were associated with depressive disorders in the multivariate model (OR = 0.82, *p* = 0.002) as was female sex (OR = 1.65, *p* = 0.007). Moreover, low vitamin B_12_ levels were linked to schizophrenia spectrum disorders (SSD, OR = 0.9982, *p* = 0.024). In contrast, higher folate (OR = 1.15, *p* < 0.001) and vitamin B_12_ levels (1.0024, *p* = 0.002) as well as female sex (OR = 7.86, *p* < 0.001) were associated with eating disorders. Insufficient or low B_12_ levels could help predict depressive and SSD respectively in child and adolescent psychiatric inpatients. Further study could help us better understand the impact of this insufficiency during the neurodevelopmental period and the potential benefits of nutritional interventions.

## Introduction

Nutritional deficiencies are major contributors to impaired neurodevelopment [1]. Early growth restriction is associated with impaired cognition [2], academic and socioemotional problems during childhood [3] and profound adverse human, social and economic consequences over the course of one’s life [4].

Vitamins B_9_ (folate) and B_12_ (cobalamin) are water-soluble B-vitamins which are closely connected to nutritional deficiencies [1]. Folate is often obtained mostly from cereals, grains, fruits, and leafy green vegetables. It is an essential component in cell division through amino and nucleic acid synthesis and plays a key role in growth and neurodevelopment [5]. Vitamin B_12_ is naturally found in animal-based foods (meat, milk, fish, and eggs) [6]. It is of key importance in the central nervous system metabolism and maintenance by preserving the myelin sheath, and in neurotransmitter synthesis. Moreover, both folate and vitamin B_12_ play key roles in several fetal and child neurodevelopmental processes, through DNA methylation and methionine synthesis [7, 8].

Although brain development begins just a few weeks after conception, the fetal and postnatal periods are times of high vulnerability due to rapid changes which require more B-vitamins to ensure proper neurodevelopment [9]. Moreover, during childhood and adolescence, several crucial neurodevelopmental processes occur [10] such as synaptic pruning and restructuration, the development of learning and memory circuits in the medial temporal lobe, executive functions in the prefrontal cortex, and myelinization processes, particularly during the transition to puberty [11].

Folate and vitamin B_12_ deficiencies lead to damage in the myelin sheath, causing myelopathy, neuropathy and neuropsychiatric disorders [12]. The demyelization induced by folate and vitamin B_12_ deficiencies can result in delayed cognitive development [7].

Regarding psychiatric disorders, in adults, low folate levels and deficiencies have been linked to depression [13], schizophrenia [9, 14, 15], first-episodes of psychosis [16], Autism Spectrum Disorders (ASD), and Attention Deficit Hyperactivity Disorder (ADHD) [10]. Also, low folate levels in serum have shown a correlation with the severity of depressive symptoms [17]. On the other hand, higher rates of vitamin B_12_ deficiency have been observed in patients with schizophrenia compared to controls [9, 18]. Vitamin B_12_ deficiency has also been associated with a two-fold higher risk of developing severe depression in adults [7, 19]. Nevertheless, not all of the studies in adults have found a relationship between low folate levels and depression [20].

In children and adolescents with psychiatric disorders, few studies have focused on folate and vitamin B_12_ levels. In a systematic review of water-soluble vitamins in this population, meta-analyses showed significantly lower vitamin B_12_ levels in ASD and ADHD patients vs. healthy controls (HC), while folate levels were higher in ADHD patients vs. HC [21]. Inconsistent results have been found regarding depressive disorder in minors, with lower folate levels in female patients vs. HC in one study [22], but not in another [23]. Looking at vitamin B_12_, a meta-analysis found no differences between children and adolescents with depression and controls [24]. Nevertheless, deficient levels of folate or B_12_ were found in 11.2% and 30.3% of children and adolescents with depressive disorders, while neither deficiency was found in HC [23]. In contrast, adolescents with anorexia nervosa tended to have high levels of folate and vitamin B_12_ in some studies [25, 26].

Overall, despite the potential impact of folate and vitamin B_12_ levels on the developing brain of children and adolescents with psychiatric disorders, few studies have been published about this issue.

The main aim of the present study was to describe serum levels of folate and vitamin B_12_ in child and adolescent psychiatric inpatients, focusing on potential links between insufficiency and deficiency status, and different psychiatric diagnostic categories. A secondary aim was to examine whether patients’ vitamin levels could be used as predictive factors associated with any of the psychiatric disorder categories in our sample.

Taking into account the previous reports in children and adolescents as well as in adults, the hypotheses of the study were, first, that child and adolescent psychiatric inpatients would present mean folate and B_12_ serum levels below the normal range, with an important prevalence of folate and vitamin B_12_ insufficiencies. Secondly, we speculated that there would be significant differences among diagnostic categories, with patients diagnosed with depression and psychotic disorders having the lowest levels and patients with eating disorders presenting the highest levels of both vitamins compared to the other diagnostic categories. Thirdly, we expected to find that some of the insufficiencies would be associated with at least one of the diagnostic categories.

## Methods

### Design, sample and assessment

A cross-sectional study was conducted including all child and adolescent psychiatric inpatients admitted during a 3-year period (01/01/2015 to 31/12/2017) at the Child and Adolescent Psychiatry and Psychology Department, Hospital Clínic, Barcelona, Spain. Exclusion criteria were readmission during the referred period and supplementation with folate, B_12_ or multivitamins up to six months prior to admission. The study was conducted according to the Declaration of Helsinki. It was approved by the ethics committee of Hospital Clínic de Barcelona (HCB/2018/0063).

### Data collection

For each patient, we included sociodemographic variables (age, sex), main psychiatric diagnoses according to DSM-5 [27], non-psychiatric comorbidities, and serum levels of folate (ng/ml) and vitamin B_12_ (pg/ml). These measurements are included in the routine protocol for blood testing in our ward and are taken the first business day after a patient’s admission.

Diagnoses were grouped into 7 diagnostic categories: (1) schizophrenia spectrum disorders (SSD): psychotic disorder not otherwise specified (NOS), schizophrenia, schizoaffective disorder, and schizophreniform disorder; (2) bipolar disorders: bipolar I and II disorders and BD NOS; (3) depressive disorders: depressive disorder NOS, major depressive disorder (MDD), MDD with psychotic features, adjustment disorder with depressed mood, and dysthymic disorder; (4) disruptive behavior disorders: conduct disorder and oppositional defiant disorder; (5) autism spectrum disorders; (6) eating disorders: anorexia nervosa and eating disorder NOS, and (7) others (including obsessive, conversive, dissociative and post-traumatic stress disorders). When comorbid psychiatric diagnoses were present, the main diagnosis leading to the current psychiatric admission was taken to be the main diagnosis (e.g. if a patient with a cannabis use disorder was admitted due to a psychotic episode, the psychotic disorder was registered as the main diagnosis).

### B-vitamin assessment

Folate and vitamin B_12_ status were assessed by measuring serum levels using an electrochemical-immunoluminescence automatized immunoassay system, which was provided by Advia-Centaur (Siemens-Healthcare, Barcelona, Spain). The reagents used were supplied by the same manufacturer. The intra-assay coefficients of variation (CV) for B_12_ were < 7.9% and for folate < 4.3%. The intra-assay CV for folate and B_12_ were < 7.8% and 9.8% respectively. External quality control was performed as per the quality-control program from the “Sociedad Española de Bioquímica Clínica y Patología Molecular”.

There is no “gold standard” test to define B-vitamin deficiencies. Given the variety of methodologies used and technical issues, it is not possible to define deficiency with definitive cut-off points [28]. According to the WHO guidelines, a serum folate level ≤ 3 ug/l (< 7 nmol/l) and vitamin B_12_ levels of < 200 ng/l (148 pmol/l) are indicative of deficiency [29]. Insufficiency has been defined by Lamers [30] as serum folate levels in the range of 3.01 to 4.9 ng/ml and B_12_ from 200 to 299 pg/ml, with levels below these corresponding to deficiency. Following this standard, we established folate < 5 ng/ml and B_12_ < 300 pg/ml as the cutoff points for insufficiency in this study.

Body stores of folate and B_12_ differ considerably. Folate’s hepatic stores provide supplies for 3–4 months, so that, depending on the daily needs of folate, a deficiency may develop within a month of poor dietary intake [28]. On the other hand, B_12_ is the best stored of all vitamins. B_12_ stores are sufficient to meet physiological needs for more than 3 years of poor dietary intake [6, 31]. Taking into account the longevity of the body’s B-vitamin storing mechanisms, patients that had undergone B-vitamin supplementation up to six months prior to admission were excluded.

### Statistical analysis

Two-sample t tests and χ^2^ tests (and Fisher’s exact test where appropriate) were used to assess differences in means and differences in proportions, respectively. Folate and vitamin B_12_ levels were compared between the groups using the median because they were not normally distributed, and non-parametric tests (Mann–Whitney U and Kruskal Wallis H tests) were used in the analysis. Median was described including the percentiles: [25th percentile, 75th percentile]. Logistic regression analysis was used to study the effects of socio-demographic variables, as well as folate and B_12_ levels, insufficiencies and deficits in predicting the outcome (psychiatric diagnostic category). The effect of different variables on the outcomes that showed a statistically significant effect on survival in univariate analyses were entered in a multivariate model, using a backward stepwise selection to obtain the final model where at each step the least significant variable was discarded until all variables in the model reached a P-value below 0.10. We computed the Hosmer-Lemeshow goodness-of-fit test for a generalized linear model fitted to binary response for the final models. To report the effect size, the odds ratio (OR) and 95% confidence interval (CI) were calculated. All P-values are two-sided and considered statistically significant if < 0.05. Data were analyzed with R software version 4.3.1 (R project for statistical computing, Vienna, Austria) and IBM SPSS version 23.

## Results

### General characteristics of the sample

A total of 729 inpatients were included for analysis. The majority of patients were female (*N* = 442, 60.6%), with a mean age of 15.1 years (± 2). Female inpatients were older than males (15.4 ± 1.7 vs. 14.8 ± 2.4 years; t=-4.042, *p* < 0.001). The sample presented 30.5% of non-psychiatric comorbidities. The most prevalent psychiatric diagnosis categories were depressive (*N* = 180, 24.7%) and disruptive behavior disorders (*N* = 149, 20.4%), and the least prevalent were bipolar disorders (*N* = 30, 4.8%) in the whole sample (Table 1).

**Table 1:** Sociodemographic and clinical characteristics of the sample

	Inpatients, whole sample (*N* = 729)	Female Sample (*N* = 442)	Male sample (*N* = 287)	t/χ2/Z ^1^	*p*
**Sociodemographics**					
Female (N;%)	442 (60.6)				
Age (years) (mean ± SD)	15.1 ± 2	15.4 ± 1.7	14.8 ± 2.4	-4.042	< 0.001
**Clinical characteristics**					
Medical comorbidities: N (%)	222 (30.5)	142 (32.1)	80 (27.9)	1.486	0.128
Obesity/overweight	23 (3.2)	15 (3.4)	8 (2.8)	0.644	0.523
Ferritin deficiency	22 (3.0)	18 (4.1)	4 (1.4)	5.673	0.017
Iron deficiency anemia	18 (2.5)	16 (36)	2 (0.5)	7.269	0.006
Atopic dermatitis	11 (1.5)	5 (1.1)	6 (2.1)	0.614	0.543
Asthma	8 (1.1)	4 (0.9)	4 (1.4)	0.165	0.731
Hypothyroidism	7 (0.9)	6 (1.4)	1 (0.3)	2.368	0.249
Psychiatric Diagnoses: N (%)					
Depressive Disorders	180 (24.7)	124 (28.1)	56 (19.5)	6.828	0.011
Disruptive Behavior Disorders	149 (20.4)	67 (15.2)	82 (28.6)	19.252	< 0.001
Eating Disorders	122 (16.7)	109 (24.7)	13 (4.5)	50.607	< 0.001
Psychotic Disorders	117 (16.0)	67 (15.2)	50 (17.4)	0.662	0.470
Autism Spectrum Disorders	64 (8.8)	13 (2.9)	51 (17.8)	47.780	< 0.001
Bipolar Disorders	35 (4.8)	20 (4.5)	15 (5.2)	0.187	0.724
Other disorders	62 (8.5)	42 (9.5)	20 (7)	1.435	0.277
**Folate and vitamin B**_**12**_ **results**					
Folate levels (ng/mL)[25th percentil, 75th percentile]*	5.3 [3.9, 7.5]	5.4 [3.9, 7.7]	5.2 [3.9, 7.4]	-0.861	0.389
Folate insufficiency: N (%)*	305 (42.9)	182 (42.4)	123 (43.6)	0.099	0.757
Vitamin B_12_ levels (pg/mL) [25th percentil, 75th percentile]**	401 [323, 502]	402.5 [327.3, 509.8]	397 [316.5, 485]	-1.373	0.170
Vitamin B_12_ insufficiency: N (%)**	138 (19.4)	78 (18.2)	60 (21.1)	0.877	0.834
Folate and vitamin B_12_ insufficiencies: N (%)***	85 (12.2)	45 (10.8)	40 (14.3)	1.846	0.194
Folate insufficiency or vitamin B_12_ insufficiency: N (%)***	352 (48.3)	211 (50.8)	141 (50.4)	0.016	0.938

Abbreviations: SD: standard deviation; N: number

^1^=Comparison between female and male samples

*=711patients (429 females and 282 males); **=713 patients; ***= 696 patients

### Folate and vitamin B_12_ levels

In the whole sample, the median value of folate was 5.3 [3.9, 7.5]ng/ml, and for vitamin B_12_ 401 [323, 502]pg/ml. The prevalence of folate insufficiency was 42.9%; vitamin B_12_ insufficiency 19.4%; and concurrent folate and vitamin B_12_ insufficiencies was 12.2%. No differences were found between males and females in terms of median vitamin levels nor in the frequency of insufficiencies (Table 1).

Inpatients with depressive disorders presented significantly lower median values of vitamin B_12_ and higher rate of vitamin B_12_ insufficiency compared to other diagnostic groups (Table 2). Furthermore, inpatients with psychotic disorders presented significantly lower median values of B_12_ compared to other diagnostic groups (Table 2).

**Table 2:** Sociodemographic, folate and vitamin B_12_ results comparing patients with depressive, schizophrenia spectrum or eating disorders vs. other diagnostic categories

Characteristics	Depressive disorders *N* = 180	Other diagnoses *N* = 549	Univariate OR (95% CI)	*p*-value	Multivariate OR (95%CI)	*p*-value
**Depressive disorders**						
Age (years) mean ± SD	15.5 ± 1.6	15 ± 2.1	1.13 (1.03, 1.23)	**0.009**		
Female sex, N (%)	124 (69)	318 (58)	1.61 (1.12, 2.3)	**0.009**	1.65 (1.15, 2.38)	**0.007**
Folate levels, (ng/mL)[25th percentil, 75th percentile]*	5.1 [3.7, 6.9]	5.4 [4, 7.7]	0.95 (0.9, 1.01)	0.119		
Vitamin B_12_ levels (pg/mL) [25th percentil, 75th percentile]**	380 [298, 472]	408 [332, 507.5]	0.9981 (0.9968, 0.9994)	**0.004**		
Folate insufficiency, N (%)*	84 (47)	221 (41)	1.26 (0.9, 1.77)	0.182		
Folate deficit, N (%)*	25 (14)	50 (9.4)	1.58 (0.94, 2.64)	0.081		
B_12_ insufficiency, N (%)**	46 (26)	116 (19)	1.71 (1.14, 2.56)	**0.009**	0.82 (0.72, 0.93)	**0.002**
B_12_ deficit, N (%)**	2 (1.1)	11 (2)	0.55 (0.12, 2.5)	0.439		
**Schizophrenia Spectrum Disorders (SSD)**						
**Characteristics**	**SSD** *N* = 117	**Other diagnoses** *N* = 612	**Univariate OR (95% CI)**	**p-value**	**Multivariate OR (95%CI)**	**p-value**
Age (years) mean ± SD	15.5 ± 1.9	15.1 ± 2	1.11 (1, 1.24)	0.053		
Female sex, N (%)	67 (57)	375 (61)	0.85 (0.57, 1.26)	0.416		
Folate levels, (ng/mL)[25th percentil, 75th percentile]*	5.2 [3.8, 7.3]	5.3 [3.9, 7.6]	0.96 (0.89, 1.03)	0.241		
Vitamin B12 levels (pg/mL) [25th percentil, 75th percentile]**	380 [325, 460]	406.5 [322, 505.5]	0.9982 (0.9967, 0.9998)	**0.024**	0.998 (0.996, 0.999)	**0.024**
Folate insufficiency, N (%)*	54 (47)	251 (42)	1.22 (0.82, 1.82)	0.337		
Folate deficit, N (%)*	13 (11)	62 (10)	1.58 (0.94, 2.64)	0.773		
B_12_ insufficiency, N (%)**	22 (19)	92 (17)	0.98 (0.59, 1.63)	0.947		
B_12_ deficit, N (%)**	2 (1.7)	11 (1.8)	0.94 (0.21, 4.32)	0.941		
**Eating disorders**						
**Characteristics**	**Eating disorders** *N* = 122	**Other diagnoses** *N* = 607	**Univariate OR (95% CI)**	**p-value**	**Multivariate OR (95%CI)**	**p-value**
Age (years) mean ± SD	15.1 ± 2.1	15.3 ± 1.5	1.04 (0.94, 1.15)	0.461		
Female sex, N (%)	109 (89)	333 (55)	6.9 (3.8, 12.53)	**< 0.001**	7.86 (4.08, 15.16)	**< 0.001**
Folate levels, (ng/mL)[25th percentil, 75th percentile]*	6.5 [4.8, 8.8]	5.1 [3.8, 7.1]	1.17 (1.1, 1.24)	**< 0.001**	1.15 (1.07, 1.23)	**< 0.001**
Vitamin B_12_ levels (pg/mL) [25th percentil, 75th percentile]**	460 [361.8, 555.5]	394 [316, 484]	1.003 (1.002, 1.004)	**< 0.001**	1.002 (1.001, 1.004)	**< 0.001**
Folate insufficiency, N (%)*	31 (26)	274 (46)	0.42 (0.27, 0.65)	**< 0.001**		
Folate deficit, N (%)*	3 (2.6)	72 (12)	0.19 (0.06, 0.62)	**0.006**		
B_12_ insufficiency, N (%)**	10 (8.8)	128 (21)	0.35 (0.18, 0.7)	**0.003**		
B_12_ deficit, N (%)**	2 (1.8)	11 (1.8)	0.95 (0.21, 4.36)	0.952		

*N = 711 patients; **=713 patients

In contrast, inpatients with eating disorders presented significantly higher median values of folate and vitamin B_12_, and lower rates of folate insufficiency, vitaminB_12_ insufficiency, and folate deficit compared to other diagnostic groups, as shown in Table 2.

### Factors associated with psychiatric diagnostic categories

**Fig. 1:**
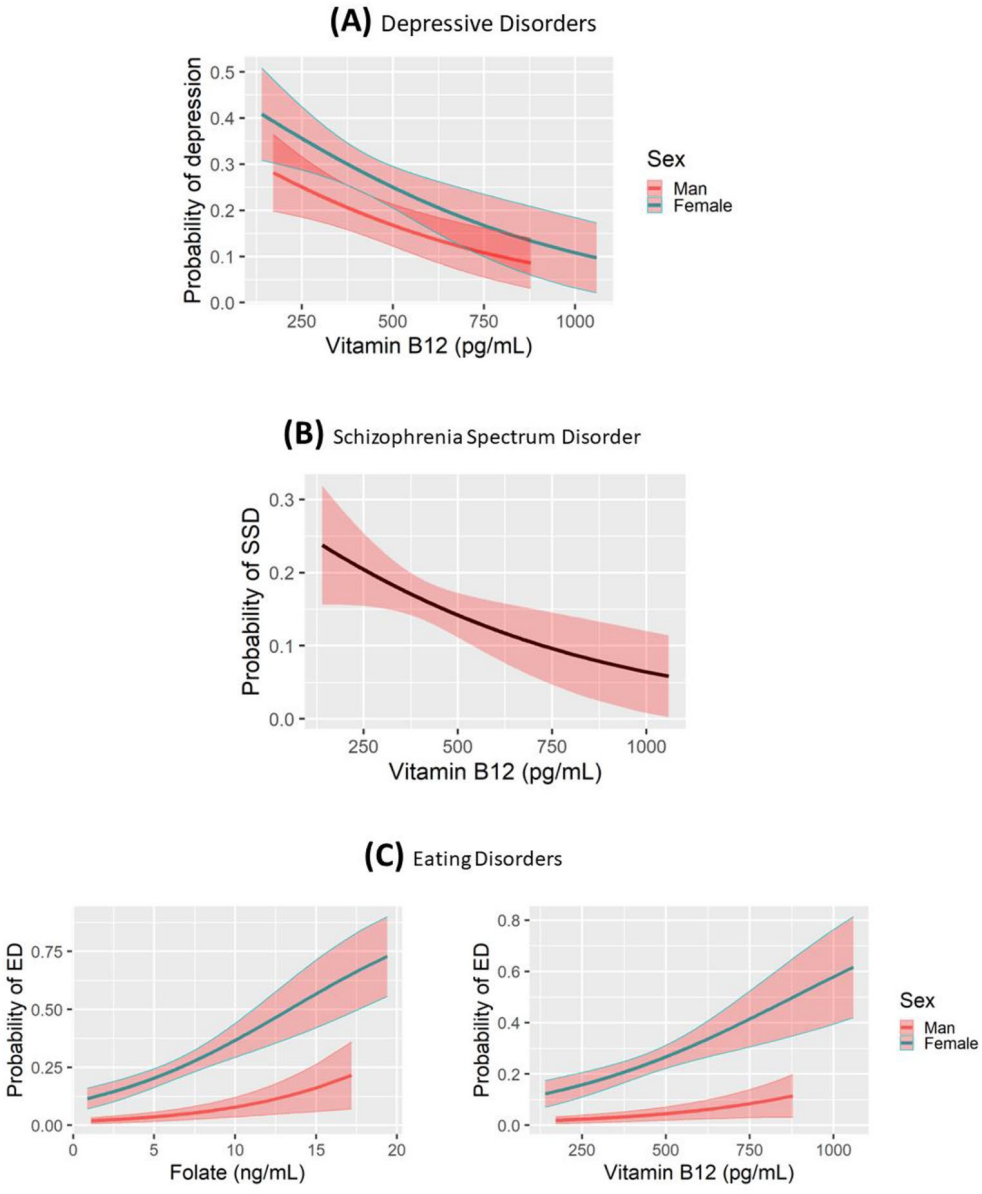
Adjusted probability prediction for a depressive disorder (**A**), Schizophrenia Spectrum disorders (**B**) and Eating disorders (**C**) according to vitamin B_12_ levels, folate levels and sex (one variable or another, depending on the diagnostic category), in our sample

Vitamin B_12_ insufficiency (OR = 0.82, *p* = 0.002) and female sex (OR = 1.65, *p* = 0.007) were associated with depressive disorders, as is described in Table 2. Figure 1A shows the adjusted probability prediction for depressive disorders regarding the significant variables for the logistic regression model, the vitamin B_12_ insufficiency and sex.

Table 2 shows the comparison in the univariate model for patients with SSD vs. the other diagnostic categories. Low vitamin B_12_ levels were associated with SSD (OR = 0.9982, *p* = 0.024) (Fig. 1B).

High folate levels (OR = 1.15, *p* < 0.001), high vitamin B_12_ levels (OR = 1.0024, *p* = 0.002), and female sex (OR = 7.86, *p* < 0.001) all predicted an eating disorder in our sample, as described in Table 2; Fig. 1C.

## Discussion

Our study found that female sex and vitamin B_12_ insufficiency were associated with depressive disorders among child and adolescent psychiatric inpatients. Also, low vitamin B_12_ levels were associated with inpatients with SSD. At the same time, female sex and high levels of folate and B_12_ were associated with eating disorders.

In our sample of child and adolescent inpatients, almost half of these subjects (42.9%) presented folate insufficiency, while 19.4% had B_12_ insufficiency and around 12% showed insufficiencies for both of these vitamins. Epidemiological studies have revealed that insufficient levels of folate and vitamin B_12_ can have adverse health effects [30] such as an increased risk of cognitive impairment [32] and chronic diseases such as cardiovascular disease [1], stroke [33] and cancer [34], apart from psychiatric conditions [13, 18]. The identification and management of folate and vitamin B_12_ insufficiency is, thus, a matter of great importance. This is especially true at young ages both because this is when neurological development occurs, and also because vitamin insufficiency is more prevalent than clinical deficiency [30]. It has been reported that across Europe 35% of adolescents of the general population presented folate insufficiency and 15% deficiency while only 2% had low vitamin B_12_ levels [35, 36], though this contrasts with a previous review of European studies which found no folate or B_12_ deficiencies in blood levels, but did not report data regarding insufficiencies [37]. Interestingly, no clear regional differences in vitamin intake have been observed in adolescents across Europe [37].

Depression was the most frequent diagnostic category in our sample, and vitamin B_12_ insufficiency was associated with this outcome. Patients with depressive disorders presented the highest rates of vitamin B_12_ insufficiency (26%), and their rates of folate insufficiency (47%) were also among the highest. Typical depressive episodes are often accompanied by anorexia and weight loss or, in the case of atypical depression, by an increase in appetite, weight gain, an unhealthy and unbalanced diet, and also hypersomnia and metabolic disturbances [38]. In both cases, depressive episodes lead to nutritional turmoil, which may help explain our results. In adults, a meta-analysis found a significant, although small, effect size in which patients with depression had lower folate levels than those without depression [13]. In children and adolescents, a systematic review described no differences in vitamin B_12_ intake between patients with depression and controls, but higher vitamin B_12_ intake was associated with lower risk of depression with an OR = 0.79; 95% CI: 0.63–0.98, *p* = 0.004) [24]. Moreover, a recently published retrospective case series described 12 adolescents with vitamin B_12_ and folate deficiency along with increased homocysteine levels who were admitted to a child and adolescent psychiatry clinic, mainly due to depression and anxiety symptoms [39]. Interestingly, in adults, supplementation with vitamin B_12_ early enough can delay the onset of depression and improve the effect of antidepressant drugs when used as adjunctive therapy [40]. Randomized control studies have also shown improvements with B_12_ vitamin supplementation in adults with depressive disorders [41].

Regarding SSD, we found an association between low vitamin B_12_ levels and these disorders in our sample. This contrasts with the findings of one study which also examined adolescent inpatients but found no insufficiencies or even differences among folate and vitamin B_12_ levels between subjects with SSD or mood disorders (including BD) and HC [42]. In adult studies, lower levels of vitamin B_12_ in patients with SSD compared to HC have been described [9, 18]. Additionally, supplementation with folate and vitamin B_12_ have been found to be beneficial, especially in improving negative symptoms, among patients with specific genetic variants in the folate metabolic pathway such as the low functioning variant of the MTHFR gene (677T) [43].

In accordance with previous studies and our hypotheses, patients with eating disorders were the subgroup with the least folate and vitamin B_12_ insufficiencies, showing the highest mean values for both vitamins. In fact, high folate and B_12_ levels were associated with eating disorders in our sample. This is consistent with other studies of water-soluble vitamins in children and adolescents with anorexia nervosa, which also found vitamin B_12_ and folate in the reference range in most of the sample [25, 44]. Castro et al. (2004), after examining vitamin B_12_, folic acid and red blood cell folate levels before and after refeeding in adolescents with anorexia nervosa, found only low red blood cell folate levels before renutrition, with B_12_ and folic acid levels within the reference range [45]. Folate and B_12_ levels in the normal range such as what we observed in our sample could also be due to a high intake of vegetables and fish by patients with eating disorders before admission as these are considered low calorie foods by most patients [46, 47]. Moreover, the long period during which vitamin B_12_ is stored in the liver until its depletion could be another possible reason for our findings [31].

Finally, female sex was associated with both depressive and eating disorders in our sample. Female adolescents have previously been found to be at higher risk of depressive symptoms and disorder than male adolescents in a meta-analysis [48]. Eating disorders are also known to be more prevalent among females [49]. Both genetic vulnerability and environmental factors could help explain the differences in the prevalence of these disorders between genders [50–52]. At the same time, it has been reported that sexual hormones could also play a role in triggering depressive disorder [53].

One possible mechanism which could link both vitamin B_12_ and folate with depressive and psychotic disorders relates to their role in the one-carbon metabolism, which is implicated in diverse processes including DNA methylation, epigenetic control of gene expression, DNA synthesis, membrane signaling, maintenance of cellular redox homeostasis and detoxification capacity [54]. One-carbon metabolism has three pathways: the folate cycle, the methionine cycle and transsulfuration. Folate is involved in all of these, while vitamin B_12_ plays a role in both the folate and methionine cycles. If there is an abnormal methylation process, the amino acid homocysteine could be produced in excess and become toxic [55]. Homocysteine could damage neurons, vascular endothelium and DNA, produce mitochondrial dysfunction and apoptotic activation [39]. In fact, systematic reviews and meta-analyses found higher levels of homocysteine in adult patients with depression vs. controls [56] as well as in adults with a first-episode of psychosis vs. controls [57].

Vitamin B_12_ has also been implicated in the inhibition of presynaptic vesicular glutamate release from rat cerebrocortical synaptosomes and may reduce the strength of glutamatergic synaptic transmission, with a potential neuroprotective effect [58].

### Strengths and limitations

Limitations of our study include the fact that the measurement of stand-alone markers of folate and vitamin B_12_ status might be insufficient for the unequivocal diagnosis of B-vitamin deficiency and insufficiency. Including intra-erythrocyte folate, holotranscobalamin and homocysteine could have improved our results [59, 60]. Nevertheless, in current clinical practice, using serum cobalamin and folate for these measurements is common and has been judged to be useful [28]. Additionally, we did not assess the nutritional patterns of the patients with a questionnaire. Therefore, the influence of patients’ dietary habits on folate and vitamin B_12_ insufficiencies could not be assessed in our study. Moreover, we did not include Body Mass Index in our analysis, although vitamin insufficiencies and deficiencies have been reported to be independent of this parameter [61]. We did not include data of the prescribed medication in the sample. Although vitamin B_12_ and folate do not usually interact with the typical psychotropic drugs used in our ward, they may with H_2_-receptor antagonists, some antiepileptic drugs, and other medications [62, 63]. Finally, we did not measure the severity of patients’ psychiatric diseases during the assessment. Thus, we were not able to examine possible relationships between vitamin levels and the severity of these patients’ illnesses.

The study also had certain strengths. This includes the large sample of child and adolescent psychiatric inpatients with serum levels of vitamins, a biomarker which could be more precise than other intake methods for measuring certain vitamins [64]. Another strength is the long inclusion period (3 years), which avoids possible seasonal nutritional variations. Finally, the sample presented with different diagnostic categories, which has enabled a comprehensive subanalysis.

In conclusion, our study found that vitamin B_12_ insufficiency was associated with depressive disorders, while low vitamin B_12_ levels were associated with SSD among child and adolescent psychiatric inpatients. In contrast, patients with eating disorders had higher folate and B_12_ levels than the other psychiatric diagnostic categories. Mental health professionals should pay special attention to folate and B_12_ levels, above all in adolescents with depressive and SSD disorders, since B_12_ may play a role in the etiopathogenesis of these illnesses. Further studies in the child and adolescent psychiatric population are needed to help offer a clearer picture of the impacts of folate and vitamin B_12_ levels and insufficiencies on young patients.

## Data Availability

The data that support the findings of this study are not openly available due to ethical reasons and are available from the corresponding author upon reasonable request.
